# The Political Importance of Voluntary Work

**DOI:** 10.1007/s10699-014-9403-x

**Published:** 2015-01-04

**Authors:** Harry Kunneman

**Affiliations:** University of Humanistic Studies, Kromme Nieuwegracht 29, 3512 HD Utrecht, The Netherlands

**Keywords:** Civic meaningfulness, Epistemological complexity, Ethical complexity, Hermeneutics, Normative professionalization, Relational biology

## Abstract

This paper aims to develop a complex articulation of the civic meaningfulness of voluntary work that clarifies its political importance as a countervailing narrative pointing beyond dominant neoliberal and consumptive articulations of a good life. To start with, it sketches a hermeneutic perspective on civic meaningfulness based on the work of Paul Ricoeur. Subsequently, it introduces the ideas of ‘ethical complexity’, ‘epistemological complexity’ and ‘diapoiesis’, building on insights from critical complexity thinking and relational biology. It argues that these notions can provide a bridge between hermeneutic perspectives on meaning and values, on the one hand, and questions of meaning and values on the level of scientific and technological developments and within professional organizations, on the other. Thus a broader, more complex picture emerges of the civic meaningfulness of voluntary work in our times.

## Introduction

This paper aims to clarify the political importance of voluntary work in view of the great moral and political challenges of our time I will argue that existentially meaningful voluntary work can contribute to new articulations of a meaningful life that point beyond the dominant neoliberal narrative, which connect a meaningful life with ever growing consumptive possibilities. A good illustration of this dominant narrative is provided by the reactions to the current economic ‘crises’ by the economic and political centers of power in Western countries. These reactions are based without exception on the presupposition that the only ‘way out’ is provided by renewed and intensified economic growth, made possible both by continued technological innovation and by severe reductions in welfare provisions. As Dominelli rightfully points out in her contribution, seen in this light an exclusive focus on voluntary work as a source of biographical meaning and existential depth, runs the risk of abstracting too much from the concrete political context we find ourselves in. The current enthusiasm of many politicians for voluntary work as a partial replacement for ‘too costly’ welfare-provisions seems indeed to be motivated at least in part by a classical capitalist agenda and its characteristic abstraction of the unequal and unjust distribution of essential resources for a good life, including care. It seems however that Dominelli’s critique silently presupposes the validity of the modern social-democratic agenda, in particular its defense of the welfare state on the basis of higher taxes and a more just income-distribution. The problem with this agenda is of course that it equally depends on economic growth within a capitalist economic framework. Both a ‘right-wing’ capitalist scenario and a ‘left wing’ social-democratic program depend for their realization on future economic growth, preferably as high as possible.

Moreover, these scenarios are heavily anthropocentric. As we all could know by now, continued economic growth in the West, in combination with the continuation of the ‘spectacular’ economic growth in ‘new’ economies like China, India and Brazil, will accelerate and deepen the ecological crises which according to most ecologists will confront us in the coming decades. Taking this disquieting prospect seriously, instead of succumbing to one of the many contemporary forms of ignoring or denying it, implies the necessity to rethink and re-articulate the framing of social progress in terms of ever decreasing poverty and continuous growth of consumptive richness for all human beings. This is a not a simple task. From Hobbes, Locke and Adam Smith, to Kant, Rawls and Habermas, ethical issues with regard to the content of a good life and moral questions with regard to the practical realization of just institutions, are defined primarily with regard to the wellbeing of human actors, to the exclusion of non-human life forms as fellow living beings with whom we have to co-create a sustainable society on this planet. Seen in this light, the most important moral and political questions confronting us in the coming decades, can no longer be limited to the question how to realize a better life and social justice for all human beings. Instead this question has to be broadened so as to include non-human life forms as fellow living beings in our quests for a good life with and for other living beings in just institutions, to paraphrase Paul Ricoeur’s well-known formula (Ricoeur 1992). The notion of meaningfulness could be very fruitful for such a broader perspective, because it connects questions with regard to a just distribution of the necessary resources for a meaningful life with questions concerning the *content* of these resources; with questions such as ‘what makes a life really worth living?’ and ‘What could be other sources of meaning besides a permanent increase of consumptive possibilities?’

Seen in this light, a focus on the meaning and meaningfulness of voluntary work is indeed politically relevant, both with regard to questions of social justice and with regard to the wider ecological challenges of our times. Especially the idea of ‘civic meaningfulness’ is helpful here, because it connects the existential meaningfulness of voluntary work with citizenship, and thus with wider moral and political questions with regard to the contribution of individuals to the flourishing of the ‘polis’ in which their lives are situated. Thus it clearly points beyond dominant neoliberal narratives with regard to the content of a meaningful life.

However, it seems to me that the notion of politics and citizenship underlying the idea of ‘civic meaningfulness’ is not rich enough to do justice to the potential political importance of voluntary work in relation to the moral and political challenges of our times. I will argue that the idea of civic meaningfulness has to be enriched in two crucial respects:

- The domain of the political should not remain limited to civil society. In terms of Jürgen Habermas’ well known distinction between ‘system’ and ‘life world’ (Habermas 1987), the idea of civic meaningfulness focuses on the life world and on civil society as the political domain par excellence and abstracts from economic, bureaucratic and technological systems as central political ‘arena’s’ of our times.
- Just like the Greek notion of the polis excluded ‘barbarians’ from the political community, so most present day definitions of politics and the polis exclude all the other life forms with whom we share the earth from ‘our’ political community. In view of the ecological challenges facing us, our notion of the ‘polis’ we belong to and contribute to, should be extended to include other life forms on our planet.

Against this background, I have set myself the task in this paper to develop a more complex articulation of civic meaningfulness that clarifies the political importance of voluntary work as a countervailing narrative pointing beyond dominant neoliberal and consumptive articulations of a good life. To begin with, I will sketch a hermeneutic perspective on civic meaningfulness based primarily on the work of Paul Ricoeur. His work allows for a clear and philosophically sophisticated articulation of the connections between existential meaningfulness on the one hand and moral and political commitments on the other. In a second step I will go into the conceptual limitations of the hermeneutic tradition, especially the exclusion of economic and technological questions and of power relations from the realm of ‘meaning’ and ‘values’. This exclusion leads to a narrow vision of politics and the ‘polis’ that stands in the way of a clear understanding of the political importance of voluntary work in our times. In order to remedy this ‘myopia’ I then introduce the ideas of ‘ethical complexity’ and ‘epistemological complexity’, building on insights from critical complexity thinking and relational biology. I will argue that these notions provide a bridge between hermeneutic perspectives on meaning and values on the one hand and questions of meaning and values on the level of scientific and technological developments and within professional organizations on the other hand. In a last step I will then connect the civil meaningfulness of voluntary work with the civil meaningfulness of good work and craftsmanship and indicate the relevance of this connection for the ecological challenges facing us: their combination provides an articulation of a meaningful life ‘with and for others’ that points beyond dominant ‘consumptive’ articulations of a good, meaningful life.

## A Hermeneutic Perspective on Civic Meaningfulness

In this paragraph I will sketch a hermeneutic perspective on civic meaningfulness based primarily on the work of one of the philosophical giants from twentieth century hermeneutic philosophy, the French philosopher and ethicist Paul Ricoeur. Two books out of his vast oeuvre are of special interest in the context of my argument: the first part of his three-volume study *Time and Narrative*, and the book which for me forms the pinnacle of Ricoeur’s impressive oeuvre: *Oneself as Another* (Ricoeur 1984, 1992).^1^

In his influential study *Time and Narrative*, Ricoeur uses Aristotle’s notion of ‘mimesis’ to shed light on the nature of a process which in his eyes antedates and underlies all forms of hermeneutic interpretation: the telling of and listening to stories—or more generally narratives—ranging from anecdotes, jokes, and simple stories to elaborate plays, political ideologies and religious traditions. Ricoeur builds upon Aristotle’s analysis of tragedies as ‘mimesis’, that is to say tragedies as ‘models’ or ‘pictures’ of human life. According to Ricoeur we have to distinguish three different forms of mimesis that come into play when a narrative is told to a listener, or a play is performed before an audience. The *first* form of mimesis, designated as mimesis 1, consists of cultural frameworks and habits that are shared (up to a point) by the speaker and the listener(s). The plot of a narrative—for instance the tragic story of King Oedipus- needs a background against which it can stand out and make sense for the listeners. A shared range of cultural frameworks provides this meaningful background.

The *second* form of mimesis presupposes this background of more or less shared meanings. Ricoeur designates this second form of mimesis as ‘emplotment’. The concept of ‘narrative plots’ is closely connected with the notion of the main ‘protagonists’ or ‘characters’ figuring in a narrative. The plot gives unity to a narrative. It refers to what is done by and happens to the main character(s) of a story as told by a narrator, for example Sophocles describing for us how Oedipus unwittingly killed his own father, became king, married his mother, and ended his life in utter despair after finding out what he had done. Ricoeur designates the plot of narratives also as a specific ‘configuration’ of possible experiences of human actors.

The notion of configuration sheds light on the *third* and last form of mimesis, which is performed by the listeners. This third form of mimesis resonates strongly with Gadamer’s influential notion of ‘hermeneutic application’ (Gadamer 1989). The hermeneutic reception of a plot involves an active reception of existential and moral meaning. By interpreting the plot of a narrative against the background of shared cultural frameworks and their own life history in the light of the existential questions and moral challenges of their daily lives, readers or listeners *re-configure* the plot as told. By thus actively receiving meaning, they both continue and transform the narrative involved. Sigmund Freud’s new interpretation of the tragedy of King Oedipus in terms of unconscious sexual desires for instance and the application of this interpretation in the context of psychoanalytic practice, would have been inconceivable for the Renaissance humanists rediscovering in awe the text of the play Oedipus Rex by Sophocles. Nevertheless, present-day Western intellectuals can perceive Freud’s transformation of the meaning of the narrative of Oedipus in the context of psychoanalytical therapies as a meaningful continuation of the same narrative content, that can moreover help them to shed light on their own life stories, for instance the painful role played by shame and guilt in their lives.

Along these lines a continuous series of ‘feed-back loops’ comes into play between the three forms of mimesis: by understanding the plots of narratives against the background of shared cultural frameworks and actively interpreting and ‘re-configuring’ the meaning of these plots in the light of the existential questions and moral challenges of their daily lives (‘applicatio’), human actors can influence and change taken for granted cultural frameworks and the models for a meaningful life contained in them.

### The Narrative Development of Identities

Against this background I can now proceed to an important innovation of the hermeneutic tradition brought about by Ricoeur: the introduction of the idea of the narrative development of identities. This idea takes central stage in one of Ricoeur’s most influential books, *Oneself as Another*, first published in Paris in 1990. In this book he sets himself the task to develop a notion of the identity of persons based on ‘the ethical perspective’, which he defines as follows: “Aiming at the good life, with and for others in just institutions” (Ricoeur 1992, p. 180). This definition divides the broad domain of ethics into two interconnected fields. The first is the field of ‘existential ethics’, dominated by the question how to live a good life. At first sight this question seems to refer to individual experiences and interpretations of a good and meaningful life and to *individual* plans and individual actions aimed at realizing a good life. However, upon closer analysis it becomes clear according to Ricoeur that reciprocal relations with other persons are of decisive importance for living a meaningful life. Following Aristotle, Ricoeur refers to genuine friendship as a paradigmatic example of an interpersonal relation that contributes to the realization of a good life. This relation brings out ‘the best’ in both of them: friends enable each other not only to experience ‘goodness’ by receiving it from the other, but also by giving it to the other, in the form of attentiveness, care and ‘solicitude’ as Ricoeur says (Ricoeur 1992, p. 192)

At this point Ricoeur introduces an insight that is of crucial importance for a philosophical clarification of the idea of civic meaningfulness. The mutual solicitude that characterizes genuine friendships connects the field of existential ethics with the second ethical field, which concerns moral justice on the level of institutions. This field of ‘institutional’ or ‘political’ morality’ brings in another ethical criterion. This criterion presupposes the criterion of a good life, but adds the idea of an equal and just *distribution* of the *resources* needed for a good life. According to Ricoeur the concept of distribution must not be limited to the economic plane, as a complement to production: “It denotes a feature fundamental to all institutions, to the extent that they govern the apportionment of roles, tasks, and advantages or disadvantages between the members of society.” (Ricoeur 1992, p. 200). According to Ricoeur, equality is to life on the level of institutions what solicitude is on the level of interpersonal relations. On the level of personal relations—such as relations between friends or between voluntary workers and those they care for—we have a unique other person before us who has a ‘face’, in the sense of Levinas, and at the same time *we* have a face as a unique person for the other. Equality on the level of institutions however is applicable to *all*. It brings in the moral horizon of universal justice:

Equality provides to the self another who is an each...the sense of justice takes nothing away from solicitude; the sense of justice presupposes it, to the extent that it holds persons to be irreplaceable. Justice in turn adds to solicitude, to the extent that the field of application of equality is all mankind. (Ricoeur 1992, p. 202)

### Idem and Ipse

By thus distinguishing between existential ethics and moral questions concerning the just or unjust distribution of necessary resources for a good life at the institutional level of the polis, Ricoeur can firmly connect them and thus bridge the notorious gap between teleological and deontological approaches in contemporary ethics. Moreover—and even more important in the context of my argument—he also succeeds in anchoring this connection in a subtle analysis of the narrative development of identities. This development is based on the mediation between two ‘poles’ of the identity of persons, corresponding with two different ways of being the ‘same’ person. Ricoeur designates these two poles with the Latin words ‘idem’ and ‘ipse’, literally meaning ‘the same’ and ‘self’. The identity of persons as idem concerns their ‘character’, understood as their characteristic way of being in the world, that is to say: all that remains the same in the flux of time and makes them recognizable for others. The idem-pole of identity thus refers to sameness as a specific, ‘repetitive’ form of permanence in time. It concerns habitual ways of acting and reacting and characteristic ways of expressing; in short all that can be confidently *predicted* with regard to specific individuals by others who know them well enough.

Ricoeur further elucidates this distinction between the idem-pole and the ipse-pole of identities in terms of the difference between ‘what’ and ‘who’. Describing the identity of persons with reference to the idem-pole of their identity, their ‘character’, answers the question *what* they are: what is more or less repetitive in their actions and reactions and thus can be confidently predicted about them. In contrast, the ipse-pole of the identity of persons cannot be described in this ‘epistemic’ way, because it concerns the question *who* they are. According to Ricoeur, answers to the question ‘who am I?’ and “who are you for me?’ refer to a different form of permanence in time that does not rest on repetition of the same!

He gives two paradigmatic examples of the ipse-pole of identity: on the one hand keeping one’s promise and being dependable for another person who counts on one’s solicitude and care; on the other hand standing up for and being true to one’s moral convictions, also designated by him as ‘attestation’. In both these paradigmatic examples the specific form of permanence in time characterizing the ipse-pole of identity, emerges *in answer* to the needs and hopes of another person (or persons). This emergence is precarious. In many cases, this ethical form of permanence in time collapses so to say under the pressure of the idem-pole of identity; that is, under the pressure exerted by specific ‘building blocks’ of one’s character whose weight pushes one in other directions then being faithful to ones promises or daring to stand up for equality and justice and remain true to one’s moral convictions.

At this point a crucial connection comes into view between his earlier analyses of the different forms of mimesis on the one hand, and his distinctions between the idem-pole and the ipse-pole of identities on the other hand. According to Ricoeur, the tension between idem and ipse is ‘mediated’ by narrative constructions and reconstructions of our identity. By reinterpreting and reconfiguring repetitive narratives that we tell to others and to ourselves about our identity, we can ‘rewrite’ up to a point the fixed building blocks of our identity that impede us to show solicitude to others, keep our promises to them or stand up for and remain true to our moral convictions. In answer to the question who we are, posed to us by others who appeal for our help and put their trust in us, we can change our identity up to a point. We can answer the question ‘who’ we are by re-telling and re-writing our life-history on the basis of answering the appeal of others for our solicitude and our moral commitment: “Here is where I stand”, or “This is the plot of my life story” (Ricoeur 1992, p. 168).

The idea of the narrative development of our identity thus functions as a bridge between the idem-pole and the ipse-pole of our identities. At decisive points in our life, we can narratively imagine different plots for the future development of our life story: as a continuation of our idem identity, or as a new figuration of our identity based on being true to the promise we made to care for others or to our moral convictions. Thus a new, ethically and morally richer plot of our life story can emerge, in terms of which we can ‘rewrite’ or reconfigure up to a point the story of our life.

## Limitations

For several reasons Ricoeur’s hermeneutic perspective on the narrative development of identities is very important in my eyes for the questions at stake in this issue, in particular the idea of the civic meaningfulness as articulation of the political importance of voluntary work. With the help of his perspective the idea of existential meaningfulness can be connected with the Aristotelian ethical tradition and understood in terms of a good life with and for others. Moreover his distinction between the idem- and the ipse-pole of identities and their narrative mediation, allows for an illuminating understanding of the development of identities. This development can now be understood as a new ‘emplotment’ of one’s life history, in which fixed or ‘frozen’ elements of one’s ‘character’ become ‘unfrozen’ so to speak in answer to a moral appeal of concrete others for one’s solicitude and care. In the third place, his analysis allows for a clear connection between the development of individual identities in the direction of a ’good, meaningful life with and for others’ on the one hand, and moral and political commitment to the development of just institutions on the other hand. In this way Ricoeur forges an important connection between teleological (or ‘Aristotelian’) and deontological (or ‘Kantian’) traditions in contemporary ethics. His hermeneutic framework thus provides the idea of the civic meaningfulness of voluntary work with more conceptual clarity and philosophical depth.

However, the phenomenological and hermeneutic roots of his perspective also bring a specific conceptual myopia with them. Building on a long Christian and humanistic tradition Ricoeur relegates all forms of moral indifference to the repetitive or idem-pole of identities and connects the narrative development of identities and moral commitment to institutional justice with solicitude and care. In this way he confirms and strengthens a deep split between two broad domains and the corresponding human possibilities that hinders a clear understanding of the political importance of voluntary work in our times. These domains pertain on the one hand to civil society and the life world, as the domain where a good meaningful life with and for others can flourish, supported by the narrative articulation of existential and moral values; and on the other hand the domain of economic production and interest-driven political action, dominated by ‘selfish’ calculation, objective knowledge and technical control. This split is closely connected with the long-standing opposition between the sciences and the humanities and the corresponding opposition between the ‘Two Cultures’ (Snow 1998). Moreover it confirms and strengthens an ontological presupposition shared by *both* ’cultures’ with regard to the ‘inanimate’ and inherently meaningless character of nature, as a domain reined by unchanging causal laws that can be discovered and used for technical control by human beings.

In my eyes, the humanistic and hermeneutic roots of Ricoeur’s perspective and the specific myopia connected with these roots, lead to a one-sided, narrow vision of politics and the ‘polis’ that stands in the way of a clear understanding of the civic meaningfulness and the political importance of voluntary work in our times. This vision furthers a myopic focus on moral reflection, community building and democratic deliberation in civil society as prime ‘locus’ of civic meaningfulness and of morally inspired political action that could lead to more just laws and further just institutions. Last but not least, it rests on an anthropocentric vision of justice that excludes other life forms from the ‘polis’. As a consequence it runs the risk of completely neglecting the importance of moral reflection and moral deliberation in social contexts *beyond* civil society and the institutions of parliamentary democracy, especially in the domains of economy, science and technology.

### Changing how we view Business’s Value

A clear illustration of the importance of moral reflection and deliberation in the economic domain is provided by the moral shifts within financial institutions in the US, resulting in the development of very sophisticated ‘mortgage backed securities’ that were both lawful and deceitful. The great amounts of ‘easy money’ that could be earned by investors by way of these ‘securities’ eventually led to the financial crisis that is still with us today. This crisis has elicited not only a great amount of critical debate within civil society and the institutions of parliamentary democracy, but has also stimulated moral reflection and deliberation on the level of corporations. An interesting example is provided by the following reflection—published in 2011—on the deeper causes of the crisis by Dominic Barton, global managing director of McKinsey:

The crisis and the surge in public antagonism it unleashed have exacerbated the friction between business and society. On top of anxiety about persistent problems such as rising income inequality, we now confront understandable anger over high unemployment, spiraling budgets deficits, and a host of other issues. Governments feel pressure to reach ever deeper inside businesses to exert control and prevent another system-shattering event. My goal here is not to offer yet another assessment the actions policymakers have taken or will take as they try to help restart global growth. The audience I want to engage is my fellow business leaders. After all, much of what went awry before and after the crisis stems from failures of governance, decision-making, and leadership *within companies*. These are failures we can and should address *ourselves*...Business must *lead* a shift...from what I call quarterly capitalism to ...a long term capitalism...This shift is...about rewiring the fundamental ways we govern, manage and lead corporations. It’s also about changing how we view business’s *value* and its role in society.” (Barton 2011, italics added, HK)

Although the shift Barton advocates from ‘quarterly capitalism’ to ‘long-term capitalism’ obviously is of great political importance for the future development of our world society, it is clear that the institutional structures of present day ‘liberal democracies’ are unable to exert sufficient political influence to bring about such a shift. In a recent survey by McKinsey, answered by one thousand business leaders all over the world, almost two-third of the respondents stated that the pressure from shareholders to maximize short-term profits has increased during the past years.^2^ Seen in this light, the moral resources available to business leaders themselves to withstand such pressures, become of prima importance for possibilities to further ‘a good life, with and for others in just institutions’ from *within* companies themselves. This goes even more in view of the long term ecological problems facing us. In a critical commentary on Barton’s analysis, the CEO of the multinational *Unilever* and ‘zeronaut’ Paul Polman, addresses this problematic as follows:

...the challenges of the future go beyond those arising from the financial crisis. We now know, in outline, what the future will look like. It will be a world where climate will change, water will be scarce, and food supplies will be insecure. Business has a chance to become part of the solution to those challenges. Just as we need to ensure that we do not repeat the mistakes which led to the recent banking crisis, so there is an equal imperative to face up to the realities of a world where 9.5 billion people will put enormous strain on biophysical resources. The rapidly growing populations of India, China, and Indonesia will all aspire to the lifestyles and living standards enjoyed by the Germans and the Californians. There is nothing that we can, or should, do to stop that (Polman 2011).

Both Barton’s analysis and Polman’s critical reactions illustrate the limitations of a view on politics that focuses on moral reflection and democratic deliberation in civil society as the prime road towards more just institutions, while neglecting the management and leadership of corporations as a new, very important domain of moral reflection and deliberation.

### Beyond the Opposition Between ‘Facts’ and ‘Values’

A similar line of reasoning is applicable to the vast influence of technological developments and innovations on the definition and shaping of necessary resources for a good life. A good example is provided by the growing influence of scientific and technological innovations in the life sciences, in particular the new possibilities for genetic manipulation and their growing influence in domains such as food, energy and in particular health. In the introduction to a recent book on the necessity of democratic control with regard to this influence, my colleague Peter Derkx and I describe the consequences of recent developments in the field of genomics as follows:

Developments in this field clearly illustrate the new political, social and ethical challenges flowing from the spectacular progress of the life sciences during the last decades. The new technological possibilities and social practices connected with these developments intrude into domains that for a long time have been the provenance of religion and ethics. There they touch upon deep-seated convictions and emotions. Moreover they are strongly influenced by economic and political interests. Thus they can no longer be adequately conceptualized along the lines of a more or less autonomous scientific development, based on a disinterested search for true knowledge, providing an objective knowledge base which interacts with values and interests only on the level of practical application. Especially in the life sciences, questions of scientific truth and scientific progress are getting more and more *mixed up* with questions regarding values and interests, as testified clearly by the heated debates on the possibilities, benefits and dangers of genomics research (Derkx and Kunneman 2013, p. 7)

Within the Sociology of Science and the related field of ‘Science and Technology Studies’, this entanglement of scientific and technological developments—or ‘techno-science’—with economic and political interests and with cultural values is variously designated as the ‘social construction of knowledge’; the rise of ‘Mode 2 knowledge production’; ‘the ‘contextualization of knowledge production’; or the ‘Janus face of science” (Knorr Cetina 1993; Gibbons et al. 1994; Nowotny et al. 2001; Latour and Woolgar 1986). These different articulations of the far-reaching changes in the production and social functioning of scientific and technological knowledge all point to the problematic character of the deeply ingrained opposition between ‘facts’ and ‘values’ and the concomitant effort to strictly separate the domains of ‘science’ and‘politics’ from each other.

A subtle articulation of the problematic character of the opposition between ‘facts’ and ‘values’ is provided by critical complexity thinking, developed among others by Edgar Morin, Paul Cilliers, Mario Giampietro and Sidney Dekker (Cilliers 1998; Dekker 2011; Giampietro et al. 2012; Morin 2008). The point of departure of critical complexity thinking is provided by insights from mainstream complexity theory, as developed during the past 30 years within information theory and neural network theory, by the members of the Santa Fé school and by the biologists Maturana and Varela, to name but a few components of its complex history (Holland 1998, 1995; Maturana and Varela 1992). According to mainstream complexity theory, complex adaptive systems are characterized by re-entrant interactions between many components, resulting in non-linear dynamics and emergent properties that cannot be understood nor explained by referring to characteristics of individual components. Complex systems are moreover composed of different layers or levels that bring many different reciprocal relations between parts and wholes in to play. Higher mammals for example, such as elephants, dolphins, primates and humans, are equipped with brains that form an important part of their body, but are themselves composed of vast amounts of interacting neurons, which in turn are grouped into different parts of the brain, such as the frontal lobe, the parietal lobe and the cerebellum. Because higher mammals are social animals living in densely interacting social groups, the bodies of individual mammals containing their brain and its neurons as a whole, are themselves part of different, dynamically changing social networks, for instance, as parents, sexual partners and allies in all kinds of controversies (de Waal 2005). These social networks in turn interact with their wider environment and thus are part of different ecological systems. As a consequence, complex systems have no clear borders, neither in space, nor in time. More precisely: their borders differ depending on the viewpoint and interests of the observer. For a brain surgeon operating a patient with a brain-tumor, the relevant system borders coincide more or less with the body of the patient in its present and future condition. For an orthodox psychoanalytic psychiatrist however, treating the same patient for post-operational stress, the youth of the patient and his relations with his mother and father will be of prime interest.

According to Paul Cilliers, this implies that all models we develop of complex systems, such as cells, ant colonies, brains and eco-systems, necessarily reduce the complexity of the system at stake. The only completely adequate model of a complex system would be the system itself! (Cilliers 1998). Critical complexity thinkers argue that, as a necessary consequence, scientific models of complex systems always involve normative choices. Their construction rests on pre-analytic choices, as Giampietro says, which unavoidably bring (mostly implicit) interests and values into play. Thus science, ethics and politics become intertwined (Cilliers 2013; Giampietro 2004).

### A Broader View of Politics

As transpires from my sketchy analysis of the growing importance of moral reflection and deliberation in the context of economic production, techno-science and ecology, the developments at stake here are wrought with open questions, uncertainties and controversies. These are clearly complex developments to which Cillier’s adagio applies that every model of complex systems and their characteristics necessarily involves normative choices. Epistemological and ethical questions cannot be separated when trying to understand these developments and judge their significance. This implies that we have to overcome deeply ingrained oppositions, such as facts versus values, explanation versus understanding, the sciences versus the humanities and have to rethink the underlying ontological separation between a ‘meaningless nature’, characterized by immutable causal laws on the one hand and human cultures and societies, characterized by meaning and values on the other hand. In my eyes this is one of the most pressing and fascinating philosophical and scientific challenges confronting us. This challenge has to be met in order to get beyond the one-sided focus on civil society as prime ‘locus’ of moral reflection and democratic deliberation. In my own, stumbling efforts to meet this challenge, I have found the most fruitful insights to date in critical complexity thinking and in relational biology. Building on these intellectual currents within present day science and on core ideas from the hermeneutic tradition, especially Paul Ricoeur, I will introduce two concepts—ethical complexity and epistemological complexity—that could help to overcome the deeply ingrained oppositions that stand in the way of a broader view of moral deliberation and politics and can do justice to the importance of moral reflection and deliberation in the context of economic production, techno-science and ecology.

## Ethical Complexity

The idea of ethical complexity is introduced by Paul Cilliers as a corollary of his epistemological critique of reductionist approaches in the sciences. It builds on Jean-Francois Lyotard’s critique of the ‘grand narratives’ and the power exerted by closed moral frameworks and ideologies that claim universal validity (Cilliers 2004, 2013; Lyotard 1999 (1993)) Over and against such closed frameworks and their universal claims, the idea of ethical complexity as developed by Cilliers foregrounds plurality and room for difference as core values of an ethics of complexity, aiming for an open and ‘provisional’ form of universality (Cilliers 2004, 2013). Cilliers has developed the idea of ethical complexity within the epistemological framework of critical complexity thinking. In the same way as reductionist and objectivist epistemologies reduce the epistemological complexity facing us when modeling complex systems, grand narratives and closed moral frameworks reduce the ethical complexity facing us when trying to understand and regulate human relations. According to Cilliers, neither of these reductions of complexity can be justified on rational grounds. This leads him to the conclusion that we have to acknowledge the fundamental provisionality of our cognitive and ethical models alike, and accept plurality and room for difference as guiding ‘dialogical’ values in dealing with the epistemological and ethical complexity of our world.

Although this articulation of ethical complexity has no doubt broken new and important conceptual ground, the fruitfulness of the idea of ethical complexity can be enhanced by connecting it with a hermeneutic framework. On the basis of such a combination ethical complexity can be articulated not only as an epistemological but also as an *existential* problematic, as a basic condition of our lives as embodied, emotional and narrative beings. This combination moreover allows distinguishing between ethically simple and ethically complex narrative plots. As I will argue in the concluding paragraph, this distinction helps to clarify the potential political importance of voluntary work with the help of a broader view of politics and civil meaningfulness, incorporating both voluntary and professional work.

### Autopoiesis and Diapoiesis

The effort to develop conceptual connections between (critical) complexity thinking and hermeneutics is a complex endeavor. Their respective vocabularies show fundamental tensions that have to be acknowledged in order to develop fruitful connections between them. These tensions are connected with the gap between the sciences and the humanities referred to above. They also crop up in the oppositions that implicitly or explicitly underlie many of the contributions to this issue between on the one hand meaning, values, humanity and moral commitment, and on the other hand economics, techno-science and interest driven political action. It is precisely this opposition and the concomitant tendency to focus on one side and disregard the other side, which resonates in Dominelli’s important warning that an exclusive focus on voluntary work as a source of biographical meaning and existential depth, runs the risk of abstracting too much from the concrete economic and political context we find ourselves in. In this paragraph I will introduce the notions of ‘autopoiesis’ and ‘diapoiesis’ as ideas that could help to bridge these oppositions and replace the gap between the opposite sides by a continuum. With the help of the continuum between autopoiesis and diapoiesis, ethical complexity can moreover be understood as a fundamental existential condition of human life.

The concept of ‘autopoiesis’ has been introduced by the biologists Humberto Maturana and Francisco Varela, in the context of their struggle against reductionist paradigms dominating theories about the nature of life (Maturana and Varela 1980). Their main thesis, spelled out in detail in their book *The Tree of Knowledge*, hinges on the idea that “living beings are characterized in that, literally, they are continually self-producing.” (Maturana and Varela 1992, p. 12) Autonomy is thus the most important defining characteristic of living beings, from the first cells up to dolphins and elephants: “We use the word ‘autonomy’ in its current sense; that is, a system is autonomous if it can specify its own laws, what is proper to it.” According to Maturana and Varela

the emergence of autopoietic unities on the face of the Earth is a landmark in the history of our solar system...not because autopoietic unities go against any aspect of physical phenomenology – since their molecular components must fulfill all physical laws – but because the phenomena they generate in functioning as autopoietic unities depend on their organization and the way this organization comes about, and not on the physical nature of their components...Thus if a cell interacts with molecule X and incorporates it in its processes, what takes place as a result of this interaction is determined not by the properties of molecule X but by the way in which that molecule is ‘seen’ or taken by the cell as it incorporates the molecule in its autopoietic dynamics (Maturana and Varela 1992, p. 48)

For several reasons, this statement has far-reaching implications. On the one hand, it connects the capacity to confer meaning with life itself. With the emergence of the very first cells as autopoietic entities, meaning comes into play as a new pattern of interaction on our planet. This pattern is anchored in the physical and chemical properties of cells, but also differs from them in decisive ways, because meaning is *meaning for an entity* and implies some measure of autonomy, that is the capability of specifying ‘its own laws, what is proper to it’ as Maturana and Varela famously say. On the other hand however, this analysis of autopoiesis in terms of autonomy has furthered an understanding of autopoiesis as a ‘self-centered’ accomplishment of living beings engaged in relentless competition for scarce resources, resulting in‘survival of the fittest’.

This understanding of autopoiesis is clearly manifest in the influential theory of ‘complex adaptive systems’ developed within the Santa Fé School. In a critical analysis of the views of one of its chief proponents, John Holland, I have tried to show that his theory of complex adaptive systems is based on ethically quite simple ideas of agency and knowledge (Kunneman 2013, p. 108). Holland ‘borrows’ his notion of agent and agency from main-stream economics. The ‘self’ implied in this model of being an agent in the world, is primarily engaged in surviving an unrelenting competition for scarce resources, by adapting successfully to a changing environment and outsmarting the competition. As a consequence the relational repertory of these agents is restricted to two basic forms of interacting with others: struggle for domination and strategic exchanges. The same goes for the knowledge of the world gathered by this self, including knowledge of other complex systems. This knowledge has a predictive character. It is based on observation, abstraction and logical inference, leading to generalization’s that allow reliable predictions of future situations and events.

So both in its relations to other entities and in its knowledge of the world, the self or ‘autos’ presupposed in the self-organizing activities of complex adaptive systems as analyzed by Holland, can be considered as ethically quite simple in comparison with the image of the self emerging in the work of hermeneutic thinkers like Paul Ricoeur. In his work we find an image of a self that is also capable of quite different relations with others, such as understanding, solicitude and friendship. According to Ricoeur, this self is also capable of relating to and learning from the tension between the different relational repertories it avails of, by narratively mediating between the idem-pole and the ipse-pole of its identity.

In the context of my ongoing discussion with Paul Cilliers,^3^ I have proposed to capture these other-directed forms of interaction with the notion of diapoiesis. This concept combines the idea of ‘poiesis’ with the notion of ‘dialogue’; both the intersubjective form of dialogue as analyzed in the work of influential ‘dialogical philosophers’ such as Michael Bakhtin, Martin Buber and Jürgen Habermas, and the ‘inner dialogue’ analyzed within the hermeneutic tradition in terms of interpreting and understanding texts and narratives. By combining their articulation of dialogical relations with the biological notion of ‘poiesis’, a much broader range of ‘diapoietic’ relations comes into view. Whereas both dialogical and hermeneutic thinkers focus on linguistically mediated forms of dialogical interaction, the notion of diapoiesis is meant to designate a much broader range of bodily mediated reciprocal interactions, which includes linguistically mediated forms of dialogical communication as a special case. This broadening has several advantages.

In the first place it allows for the inclusion of bodily interactions that are welcomed by both partners, such as physical support, care, caresses and consensual erotic interactions into the family of diapoietic relations. In this way the notion of diapoiesis helps to overcome the implicit anthropocentrism of dialogical and hermeneutic thinkers alike, and foregrounds the deep roots of diapoietic relations in our evolutionary history. As argued by relational biologists like Margulis, Ulanowicz, Damasio and de Waal, symbiosis, cooperation and care are pervasive and important forms of interaction that have emerged already early in the evolutionary history of life on our planet (Margulis 1981; Ulanowicz 2009; de Waal et al. 2006).

The work of Frans de Waal is of special interest here. In his eyes, interactions between humans are characterized by the combination of two completely different, but equally basic patterns of interaction. He associates the first pattern with the evolutionary heritage connecting us with the Chimpansees. This pattern is characterized by a permanent, often vicious struggle for power both at the top and at lower levels of the social pyramid, which determines the unequal distribution of food and of possibilities for having sex. The second pattern is associated by the Waal with another ancestral lineage, connected with the Bonobo’s. Their interactions follow a different, matriarchal pattern, characterized by the reciprocal exchange of sexual and erotic pleasure as the most important way for dealing with tensions and conflicts. According to de Waal, Bonobos’ also show a high level of solicitude for one another, and sometimes even attention and care for the welfare of other creatures, such as wounded birds. Because we humans combine these two very different patterns, we should be considered in his eyes as “one of the most internally conflicted animals ever to walk the earth.” (de Waal 2005, p. 237; de Waal 2009; Kunneman 2007).

Building on de Waal’s seminal work, the relational repertory of higher mammals such as elephants, dolphins, primates and humans can be pictured as a continuum between two poles:

on the one hand autopoietic relations, characterized by autonomously defined goals and competition for scarce resources by way of violent struggle or by way of strategic exchanges based on calculation and prediction;

on the other hand diapoietic relations, provisionally defined as all forms of interaction between living beings that emerge out of co-creation and reciprocally enhance their wellbeing (Kunneman 2010, p. 146).

With the help of this continuum, ethical complexity can be understood on a more fundamental level: not only in epistemological terms—as the acknowledgement of the provisional character of all ethical perspectives and values and the confrontation with difference—but also as a basic *existential* condition of human life, rooted in our evolutionary heritage. Seen in this way ethical complexity stems from the fact that our emotions, values and relations with others are distributed over a wide and conflictual continuum. They are not glued to either autopoiesis or diapoiesis, but can—at least potentially—move over all positions within this continuum: they can ‘jump’ from one pole to the other; they can oscillate between the two poles; they can ‘stick’ to one end of the continuum or the other; and on top of that we can even be pulled towards both poles at the same time, and be torn by conflicting emotions and values and the resulting ambivalent or outright contradictory relations with ourselves and with others.

This brings me to the second conceptual advantage flowing from my existential articulation of ethical complexity with the help of the continuum between autopoiesis and diapoiesis. This continuum allow for a distinction between ethically simple and ethically complex relations and narrative plots. I propose to designate as ethical simple all emotions, relations and narratives that are restricted to *either* pole of the continuum between autopoiesis and diapoiesis. In contrast emotions, relations and narratives that acknowledge and confront *the tension* between these two poles can be designated as ethically complex. With the help of this articulation of ethical simplicity and ethical complexity, it can indeed be said that the image of the self underlying Holland’s theory of complex systems and mainstream, ‘neoliberal’ economic theory, is characterized by ethical simplicity. The same goes however for all moral perspectives and narratives that picture ‘human nature’ as fundamentally dia-poietic and repress the importance of our ‘Chimpanzee-heritage’, both in themselves and in others.

### Epistemological Complexity

In this subparagraph I will introduce the idea of epistemological complexity as a second concept that helps to connect central insights from Paul Ricoeur with ideas and concepts from critical complexity thinking and relational biology. To that end I build on Cilliers’ analysis of ethical complexity referred to above and try to enrich it with ideas from two ‘relational’ biologists. In the first place Robert Rosen, especially his audacious effort to transplant Aristotle’s classic analysis of four different forms of causality to contemporary discussions on reductionist versus autopoietic explanations of life and its fundamental properties (Rosen 1991, 2000). In the second place Antonio Damasio, especially his views on the narrative character of emotions (Damasio 2003, 2010).

Building on Aristotle’s distinction between material, efficient and formal causality, Rosen argues that the properties of living beings cannot be explained solely in terms of material and efficient causes (Rosen 2000, p. 84, 158). The causal framework of Newtonian mechanics—which only allows for material and efficient causes and relegates formal and final causes to the domain of metaphysics—does not suffice to explain the ability of living beings to anticipate future states of their world. In the fall for example, many species of trees start to drop their leaves, although the temperature has not yet lowered substantially. They act in the here and now on the basis of an anticipation of a future state of their world. Rosen proposes to designate this property of living beings to develop anticipatory models of their world as a basis for action in their world as formal causality, and distinguish this form of causality clearly from material and efficient causes, which do not involve a model of the world.

Rosen’s analysis resonates strongly with the analysis of autopoiesis by Maturana and Varela and with Holland’s analysis of the properties of complex adaptive systems. Rosen’s perspective has the great advantage however of shedding light on the long-standing epistemological and methodological debates within the philosophy of science on the differences between the natural sciences and the humanities. The opposition between explanation and understanding takes central stage in these debates. Whereas the knowledge accumulating in the sciences would be based on causal explanation with the help of empirically verified natural laws and thus have an objective character, the insights gathered in the humanities would be value laden, based as they are on the culturally mediated understanding of motives, utterances and cultural achievements of human actors (Dilthey 1883,1989; Hempel 1966). As argued above, this opposition also strongly resonates in Ricoeur’s hermeneutic perspective. Seen in the light of the recent conceptual innovations within biology and complexity theory, especially Rosen’s biological reformulation of the Aristotelian formal cause, this long-standing opposition between causal explanation and value laden understanding loses its absolute character. When Aristotle’s ‘causa formalis’ is interpreted as the ability of living beings to develop anticipatory models of their world and shape their actions in the here and now accordingly, a whole range of actions of living beings can be understood and explained on the basis of the content of their anticipatory models and the emotions connected with them.

### Emotions as Narrative Plots

Rosen’s innovative analysis of the nature of formal causality helps to enrich the idea of epistemological complexity and points to a new bridge over the cleft between the sciences and the humanities. On the one hand, in the light of his analysis valid explanations of natural processes are no longer restricted to the demonstration of material and efficient causes. Anticipatory modeling by living beings is itself a natural process that has emerged in the course of the evolution of life on our planet. The explanation of natural events in terms of formal causes becomes a legitimate form of scientific explanation. On the other hand the strict epistemological and ontological division between the realm of ‘inanimate’ and ‘meaningless’ nature on the one hand and the domain of ‘meaningful’ human culture and civilization, characterized by values and hermeneutic understanding and the other hand, also loses its plausibility. Even very simple organisms such as the worm C. elegans develop simple models that allow them to understand their world in terms of what is good and bad for them and act accordingly. Neurobiologist Antonio Damasio designates such models as emotions, and even speaks of the narrative character of emotions as the evolutionary breeding ground of consciousness and conscious feelings (Damasio 1999, 2010).

At this point an interesting link comes into view with the first, hermeneutic part of my argument. Damasio’s analysis of the narrative character of emotions can be reformulated with the help of some of the hermeneutic concepts and insights sketched above. When I understand Damasio’s analysis correctly, he suggests that emotions can be understood as narratives told by the body to itself. The hermeneutic process involved here is at first primarily ‘receptive’. In hermeneutic terms emotions could be understood in terms of mimesis one and mimesis two. The (species-specific!) array of meaningful sensory impressions, provide the mimesis-one background for a range of emotions, that can be understood as mimesis two, that is as a range of specific narrative plots, such as pain, lust and aggression that are enacted by the body. Here we do not have consciousness yet; there is no mimesis-three, no interpretation of these plots in relation to other actual or possible plots and alternative courses of action. There is just ‘enactment of meaning’. As soon as consciousness emerges, and living beings start to feel their emotions, as Damasio says, active interpretation of the narrative plots conveyed by emotions emerges, against the background of past experiences and in the light of imagined possible plots. Moreover, when they acquire consciousness, living beings also become narrators. They are no longer only receiving and experiencing meaning, like the worm moving ‘eagerly’ towards food or ‘excitedly’ fleeing from danger. Moreover, on the basis of their interpretation of this meaning, they can also express their feelings in a conscious form, as an embodied narrative told as conscious actors to themselves, resulting in a ‘stream of consciousness. Moreover, by means of body language, they then become narrators for other conscious living beings, conveying simple plots, such as ‘I am in pain and need help’, ‘danger there, beware’, ‘I lust for you’, etcetera. By the same token, when living beings become narrators of existential plots, they also become interpreters of plots bodily conveyed to them by other living beings.

Along these lines, by combining Rosen’s analysis of anticipatory modeling and Damasio’s perspective on emotions on the one hand with hermeneutic insights into the different forms of mimesis on the other hand, a reinterpretation of the fourth mode of explanation distinguished by Aristotle comes into view: explaining actions with the help of a ‘causa finalis’, that is to say on the basis of the ‘telos’ involved. This is of course a form of explanation that is completely anathema within modern, Newtonian science that prides itself on the radical breach it has brought about with all forms of ‘teleological’ explanation. However, when—following the lead of Rosen, Damasio and other contemporary biologist—Aristotle’s ‘causa formalis’ is reinterpreted in terms of the ability of living beings to develop anticipatory models of their world and shape their actions with the help of these models in combination with specific narrative plots conveyed by emotions, a new interpretation of Aristotle’s ‘causa finalis’ becomes possible. The ‘causa finalis’ can now be understood in terms of the moral orientation provided by—mythical, religious, philosophical, political—narratives, anticipating a more just future on the level of social groups and political communities. As Frans de Waal has argued on the basis of his empirical research into ‘chimpanzee politics’, female chimpanzees already avail of clear moral emotions concerning actions of alpha males that are more or less beneficial for the group as a whole (de Waal et al. 2006). When human cultures emerge, the narrative plots underlying these moral emotions are articulated in the form of rituals, symbols and, eventually, in language.

This reinterpretation of Aristotle’s ‘causa finalis’ foregrounds the moral orientation or the moral ‘horizon’ provided by the anticipation of a more just future on the institutional level of group relations or political communities. The temporal dimension is crucial here. The material and efficient causes dominating modern (philosophy of) science abstract from time and history. In the Newtonian universe, the ‘arrow of time’ can point both forward and backward, as Prigogine says (Prigogine 1980). The same causal scheme can be used to predict future positions of the planets, or to ‘retrodict’ previous positions. As long as the ‘epistemological space’ is limited to explanations in terms of material and efficient causes, there is no history, no development, no meaning, but only repetition of the same, ‘idem’. As soon as life emerges on our planet however, anticipatory models come into the world, first related to the prediction of survival-relevant patterns and later, when emotions and feelings have emerged, augmented by a rapidly diversifying collection of emotionally charged narrative plots. In combination with anticipatory models concerning different efficient causes, these narrative plots provide ‘causae formales’ for individual actions. When primate cultures develop into human cultures, a new type of final causality emerges. The imaginative narrative anticipation of more just institutions provides a moral horizon that acts as ‘causa finalis’ for actions by individuals and groups contributing in *their* eyes to the development of more just institutions.

Of course, the clause ‘in *their* eyes’ is crucial here. I consider the different forms of causality—material, efficient, formal and final—as epistemological categories. They refer to different types of explaining and understanding patterns, events and possible developments in our world, and to corresponding forms of influencing them. For social animals like elephants, wolves, primates and humans the world is also, and even primarily a social world. The way in which essential resources are distributed in the groups they live in and the way in which inevitable conflicts over this distribution are handled, is of prime importance for them, as testified by the broad array of ‘social’ emotions and feelings connected with questions of distribution and power. As an epistemological category, formal causes refer to the understanding and explaining of actions of living beings on the basis of the anticipatory models and narrative plots they develop to make sense of their world. In the course of the evolutionary development of these models and narratives, a new form of anticipatory modeling emerges, characterized by the narrative imagination and anticipation of a more just future on the level of institutional relations. The appeal of the moral horizon articulated in such moral narratives, serves as causa finalis, as explanatory ground, for actions contributing to the development of more just institutions in the eyes of the actors involved. But this form of explanation does not imply an ethical endorsement of the moral horizon involved. An ethical appraisal of such a moral horizon becomes possible by connecting this explication of epistemological complexity in terms of four different forms of causality, with the ethical complexity and simplicity of narratives and narrative plots.

To this end I connect Ricoeur’s distinction between the idem-pole and the ipse-pole of identities with the notion of ethical complexity and the continuum between autopoiesis and diapoiesis. Ethically complex relations and ethically complex narrative plots are characterized by acknowledging and ‘working through’ the tension between them on different, related levels. To start with, this involves emotional and narrative ‘identity-work’, directed at acknowledging and resisting the strong pull of autopoietic emotional and cultural plots, thus opening oneself up to the appeal of concrete others. In the second place, the idea of ethical complexity involves the connection between ‘a good and meaningful life with and for others’ on the one hand, and efforts directed at more ‘just institutions’ on the other hand. As Ricoeur underlines—building on insights from the Frankfurter Schule, in particular from Habermas—this wider moral horizon of a more just society not only involves the extension of ‘solicitude’ to others whom one will never meet face to face, but also brings in another, political dimension: the dimension of a just division of essential resources for a good, meaningful life. Addressing this political dimension necessarily implies the *confrontation* with dominant power relations and the autopoietic dynamic underlying these relations of power. It involves ‘speaking truth to power’ as Foucault says (Foucault 2001). In contrast, ethically simple relations and ethically simple narrative plots, remain restricted to either pole of the continuum and disregard or suppress the reality and the importance of the other pole.

## Conclusion

I have set myself the task in this paper to develop a more complex articulation of civic meaningfulness that clarifies the political importance of voluntary work as a countervailing narrative pointing beyond dominant neoliberal and consumptive articulations of a good life. As it stands, the idea of civic meaningfulness certainly is a step in the right direction. It provides an important contribution to a moral narrative and a political vision connecting the meaningfulness of voluntary work on the level of individual relations with the contribution of voluntary work to more diapoietic relations on the level of civil society. The idea of the civic meaningfulness of voluntary work and the moral narrative connected with it, thus help to articulate a moral horizon characterized by ethically more complex narrative plots pointing beyond dominant neoliberal articulations of a good and meaningful life. Its potential political importance is hampered however because it abstracts from the economic, political and ecological systems in which civil societies are embedded. Formulated in terms of Habermas’s distinction between ‘system’ and ‘life world’ it focuses on the life world and on civil society as the breeding ground for moral and political commitments and disregards the importance of moral reflection and moral deliberation in economic, scientific and technological contexts.

Against this background I can now bring my argument to a conclusion by introducing the meaningfulness of *professional work* as a supplement to the meaningfulness of voluntary work, allowing to extend the idea of civic meaningfulness to politically meaningful work in economic, scientific and technological contexts. The idea of the meaningfulness of professional work builds on research undertaken in the last 20 years at the University of Humanistic Studies into the theory and practice of ‘normative professionalization’ (Kunneman 2005; Kunneman and Suransky 2011; Jacobs et al. 2007; Nap 2012). The idea of ‘normative professionalization’ has emerged in the nineties as a critical antidote against the growing emphasis in domains such as social work, education and care on the technical basis of professional action and on scientifically proven ‘evidence-based’ knowledge. By underlining the normative character of professional action in public and semi-public organizations, we tried to foreground the entanglement of professional knowledge and professional action with existing relations of power, but also the possibilities for developing a critical, reflexive relation towards the concrete embodiment of these power relations in professional knowledge-claims and organizational norms and regulations. Such a critical and reflexive relation rests not only on the appeal for ‘solicitude’ in relations with others, but also on the appeal of ‘good work’, as analyzed by Richard Sennett’s in terms of craftsmanship and cooperation (Sennett 2008; Kunneman 2012). Moreover normative professionalization is connected with a complexity-sensitive epistemological framework, encompassing the whole range of different forms of causal explanation and understanding. It implies confronting both ethical and epistemological complexity in view of good, meaningful work with and for others and the development of more just institutions. Normative professionalization thus involves an increase of the professional meaningfulness of one’s work. It furthers the conditions for a good, meaningful life not only on the level of personal relations and civil society, but also on the level of economic and political systems.

It is clear that these systems are themselves confronted with a steady increase of complexity, not only in their ‘environment’, but increasingly also on the level of their own inner functioning. This increasing complexity manifests itself on the level of the professional knowledge that present day organizations need so urgently to be ‘competitive’ and ‘innovative’. As David Schön states with regard to the challenges confronted by present day professionals:

In the varied topography of professional practice, there is a high, hard ground where practitioners can make effective use of research-based theory and there is a swampy lowland where situations are confusing ‘messes’ incapable of technical solution. The difficulty is that the problems of the high ground, however great their technical interest, are often relatively unimportant to clients or to the larger society, while in the swamp are the problems of the greatest human concern.” ((Schön 1983, p. 41))

For me this acute observation relates directly to the ecological challenges we face. These challenges confront us with the severe limitations of the ethically simple, autopoietic vision of unending technical progress towards total control over nature as the secure basis of unlimited growth of consumptive wealth (Wal van der 2011). In our times we urgently need ethically more complex narratives, encouraging us to venture into ‘swampy lowlands’ where we are both ‘out of control’ and can experience deeper forms of ‘civic meaning’, both on the level of personal and of professional relations.

Here at last the political importance of voluntary work comes into view. Instead of underlining the differences between voluntary work and professional work, it would be wiser to foreground the strong kinship between the moral horizon of normative professionalization and professional meaningfulness on the one hand and that of voluntary work as a source of civic meaningfulness on the other hand. They both give access to experiences and articulations of civic meaningfulness that provide alternatives for the dominant consumptive framing of a meaningful life. Thus their combination is of prime importance for confronting the great moral and political challenges of our times against the moral horizon of a more just, sustainable world society.

## Footnotes

1 This paragraph is based in part on a previous publication: Cf. Kunneman (2013, pp. 114–119).

2 Quoted by De Volkskrant, 30-12-2013, p. 1.

3 Paul Cilliers suddenly died of a brain haemorrhage in the summer of 2011. I miss him very much, both as a colleague and as a dear friend, and try to continue our fascinating conversations in writing, in honour of his brilliant pioneering work in critical complexity thinking and of the gift of his friendship.
